# Whole-Genome Sequence of an Avian Influenza A/H9N2 Virus Isolated from an Apparently Healthy Chicken at a Live-Poultry Market in Indonesia

**DOI:** 10.1128/MRA.01671-18

**Published:** 2019-04-25

**Authors:** Arindita N. Novianti, Krisnoadi Rahardjo, Rima R. Prasetya, Aldise M. Nastri, Jezzy R. Dewantari, Adi P. Rahardjo, Agnes T. S. Estoepangestie, Yohko K. Shimizu, Emmanuel D. Poetranto, Gatot Soegiarto, Yasuko Mori, Kazufumi Shimizu

**Affiliations:** a Faculty of Veterinary Medicine, Airlangga University, Surabaya, Indonesia; b Indonesia-Japan Collaborative Research Center, Institute of Tropical Disease, Airlangga University, Surabaya, Indonesia; c Center for Infectious Diseases, Kobe University Graduate School of Medicine, Kobe, Japan; University of Maryland School of Medicine

## Abstract

We isolated an avian influenza A/H9N2 virus from an apparently healthy chicken at a live-poultry market in January 2018. This is the first report of a whole-genome sequence of A/H9N2 virus in Indonesia. Phylogenetic analyses indicated that intrasubtype reassortment of genome segments is involved in the genesis of the A/H9N2 virus.

## Abstract (precis)

We isolated an avian influenza A/H9N2 virus from an apparently healthy chicken at a live-poultry market in January 2018. This is the first report of a whole-genome sequence of A/H9N2 virus in Indonesia.

## ANNOUNCEMENT

Avian influenza A/H9N2 virus was found in domestic poultry and wild birds worldwide and became one of the dominant subtypes of avian influenza virus (1, 2). The virus was isolated also from swine (3). Several cases of human infections were also reported (4, 5). A/H9N2 virus causes respiratory infection and replicates in the reproductive tract in chickens, resulting in decreased egg production (6). In addition, A/H9N2 virus provides some parts of internal genes to a new lethal reassortant in humans (7–9), such as H7H9 virus.

In Indonesia, there were A/H9N2 virus outbreaks in chickens causing decreased egg production from December 2016 to May 2017 (10). In January 2018, we isolated an avian A/H9N2 virus, A/chicken/East Java/Spg147/2018, from an apparently healthy chicken at a live-poultry market. The virus was grown in 10-day-old embryonated chicken eggs for 2 days at 37°C, and the allantoic fluid served as the virus stock. For genome analysis by next-generation sequencing, total RNA was extracted from the virus stock using a QIAamp viral minikit (Qiagen, Tokyo, Japan); linear polyacrylamide was used as a carrier instead of tRNA. An RNA library was prepared using a TruSeq RNA sample preparation kit v2 (Illumina, Japan). The library was loaded in the flow cell of the 300-cycle MiSeq reagent kit v2 (Illumina, USA). The barcoded multiplexed library sequencing (2 × 150 bp) was performed on a MiSeq platform (Illumina). The MiSeq platform generated FASTQ files in which the primer and adapter sequences were trimmed. The files were imported in CLC Genomics Workbench v8.1 (CLC bio, Japan) for analysis; the total number of reads was 929,006, the average read length was 141.5 bp, and the average of the Phred quality scores was Q34.1, 84.4% of which were over Q30 (99.9% accuracy of base calling at a particular sequence position). The total reads were filtered to remove reads with poor quality (those with <Q30, <26 bp long, or containing more than two consecutive ambiguous bases). After filtering, 630,095 reads remained. The filtered reads were mapped to the genomes of 27 reference viruses of influenza type A virus, including all subtypes of hemagglutinin (HA) (H1 to H18) and neuraminidase (NA) (N1 to N11), using the CLC Genomics Workbench. A total of 24,506 reads were mapped to the reference sequences, 78.7% of which were aligned on the genome (or 8 genome segments) of one of the reference viruses, A/chicken/Guangxi/LS/2013 (H9N2). Tentative complete 8-segment genome sequences were constructed from the assembled consensus and common sequences of type A influenza viruses at the 5′ end (12 nucleotides [nt]) and 3′ end (13 nt) of the genome segments. The filtered reads were mapped again to the tentative complete genome sequence, and 24,146 reads were aligned to the 8 segments. The Q scores of the mapped reads ranged from 34 to 39, with an average of 37.7. The assembled consensus sequences covered 99.8% of the tentative complete genome (13,605 nt), and the mean depth of coverage was 211. The genome comprised eight segments, polymerase basic 2 (PB2) (2,341 nt), polymerase basic 1 (PB1) (2,341 nt), polymerase acidic (PA) (2,233 nt), HA (1,742 nt), nucleoprotein (NP) (1,565 nt), NA (1,466 nt), matrix protein (M) (1,027 nt), and nonstructural protein (NS) (890 nt). The consensus sequences lacked 0 to 6 nt at the 5′ and 3′ ends of the 8 segments within the common end sequences.

The amino acid sequence at the HA cleavage site is PSRSSR↓GLF, which is characteristic of low-pathogenic avian influenza viruses (11). The PB2 protein had an E at position 627 and a D at position 701, which is characteristic of viruses of avian origin. However, the receptor binding site of HA had L222 and G224 (H5 numbering), which suggests that it has the ability to bind with a sialic acid-2,6-NeuAcGal linkage and might have the potential to infect humans (12).

**FIG 1:**
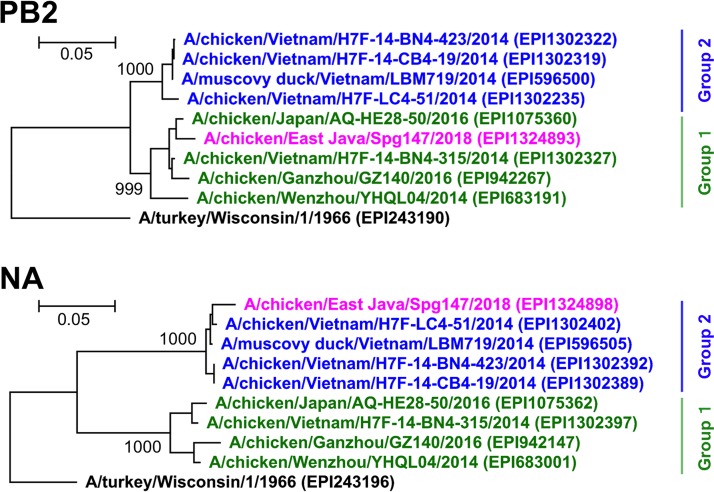
Phylogenetic analysis of PB2 and NA genome segments of A/H9N2 viruses. The phylogenetic trees were generated using coding sequences of PB2 and NA segments in the genetic information processing software Genetyx v14 (Genetyx Co., Tokyo, Japan), using the neighbor-joining method with 1,000 bootstrap replicates and the Kimura 2-parameter model. The tree was rooted from the A/turkey/Wisconsin/1/1966 virus. The viruses included were selected because either the PB2 or NA sequence was highly identical to that of our H9N2 isolate, A/chicken/East Java/Spg147/2018 (pink) by BLAST analysis in the GISAID EpiFlu database; the accession numbers are in parentheses following the virus names. The eight selected viruses formed two distinct groups in both the PB2 and NA phylogenies, which were named group 1 (green) and group 2 (blue), and the members in each group were same between the two phylogenies. PB2 of our isolate belonged to group 1, while NA belonged to group 2, indicating that intrasubtype reassortment of genome segments is involved in the genesis of the A/H9N2 virus.

BLAST and phylogenetic analyses revealed that the PB2 and NA segments of this virus were derived from different groups of H9N2 virus (Fig. 1), indicating that intrasubtype reassortment of genome segments is involved in the genesis of the A/H9N2 virus.

### Data availability.

The genome sequence of A/chicken/East Java/Spg147/2018(H9N2) has been deposited in the Global Initiative on Sharing All Influenza Data (GISAID) EpiFlu database (13) under the accession numbers EPI1324893 to EPI1324900. The raw reads of the FASTQ format will be provided by the corresponding author as requested.
